# Morphology–Transport Coupling and Dissipative Structures in PEO–PS+LiTFSI Electrolytes In-Operando Conditions

**DOI:** 10.1021/acsami.4c18838

**Published:** 2025-01-30

**Authors:** Mario Tagliazucchi, Marcus Müller

**Affiliations:** † Departamento de Química Inorgánica Analítica y Química Física, Ciudad Universitaria, Facultad de Ciencias Exactas y Naturales, Universidad de Buenos Aires, Pabellón 2, C1428EGA Buenos Aires, Argentina; ‡ Instituto de Química de los Materiales, Ambiente y Energía (INQUIMAE), Ciudad Universitaria, CONICET, Facultad de Ciencias Exactas y Naturales, Universidad de Buenos Aires, Pabellón 2, C1428EGA Buenos Aires, Argentina; § Institute for Theoretical Physics, Georg-August University of Göttingen, 37077 Göttingen, Germany

**Keywords:** block copolymer, lithium battery, polymer electrolyte, ion flux, self-assembly, polarization, simulation, salt gradient

## Abstract

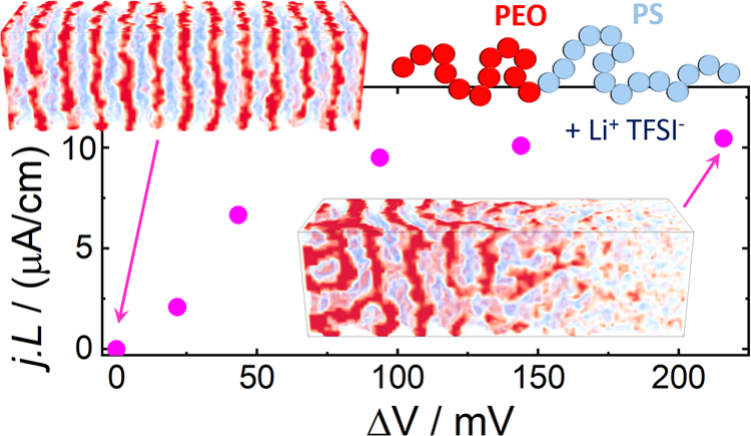

A Single-Chain-in-Mean-Field (SCMF) algorithm was introduced to study block copolymer electrolytes in nonequilibrium conditions. This method self-consistently combines a particle-based description of the polymer with a generalized diffusion equation for the ionic fluxes, thus exploiting the time scale separation between fast ion motion and the slow polymer relaxation and self-assembly. We apply this computational method to study ion fluxes in electrochemical cells containing poly(ethylene oxide)-polystyrene (PEO–PS) block copolymers with added lithium salt. Blocking of the anion fluxes by the electrodes *in-operando* conditions polarizes the cells and results in an inhomogeneous salt-concentration profile. This gradient of salt concentration triggers lamellae-to-disorder and disorder-to-lamellae transitions near the electrodes, in good agreement with previous experimental observations. The effects of the selectivity of the electrode surface, the salt concentration and the voltage applied to the cell are systematically studied. For PEO-selective surfaces, the lamellae parallel to the electrode that forms at low applied potentials transition to a bicontinuous morphology at high applied potentials in order to allow ion transport through the insulating PS layers. The formation of this dissipative structure, which is unexpected considering the equilibrium behavior of the material, is in line with the principle of maximum entropy production. In summary, the transport and morphology in PEO–PS electrolytes are strongly coupled: ionic currents influence self-assembly, which in turn modulates the ionic fluxes in the cell.

## Introduction

Polymers are an attractive alternative to liquid solvents for the formulation of battery electrolytes.^1,2^ Microphase-separated block copolymers were introduced in this context to improve the mechanical and thermal properties of polymer+salt electrolytes.^3^ A paradigmatic example of this strategy is that of the block copolymers formed by poly(ethylene oxide) (PEO) and poly(styrene) (PS) with added LiTFSI salt, i.e., PEO–PS+LiTFSI.^4,5^ In this material, the glassy PS domains grant mechanical stability and the PEO regions selectively dissolve and transport Li^+^ and TFSI^–^. The PEO–PS+LiTFSI system can adopt the same morphologies available to salt-free block copolymers;^5^ however, the occurrence of these phases strongly depends on the concentration of LiTFSI.^5^ Increasing the salt content has an effect similar to increasing the polymer incompatibility (quantified by the Flory–Huggins parameter, χ),^6^ and, thus, it can lead to a disorder-to-order transition. This effect is traced back to the selective localization of salt in the high-dielectric-constant PEO block, which favors its segregation from the low-dielectric-constant block, PS.

Electric-field-driven (migration) fluxes within a battery drive anions and cations in opposite directions. In Li-batteries, only the Li^+^ ions undergo electrochemical transformations at the electrodes, whereas the anions, TFSI^–^, accumulate near one electrode and are depleted near the other. This process continues until the diffusive flux generated by the resulting concentration gradient cancels out the migration flux.^7^ To avoid deviations from local electroneutrality, the Li^+^ concentration profile in the nonequilibrium steady state (NESS) matches that of the anion, therefore redistributing the neutral salt. Since the morphology of PEO–PS+LiTFSI system depends on salt concentration, this process generates local morphological transitions in the electrolyte, which were experimentally characterized by Balsara and co-workers using Small Angle X-ray Scattering (SAXS).^8−10^ In this work, we theoretically consider PEO–PS+LiTFSI block copolymer electrolytes *in-operando* conditions^8,9,11^ and show that ionic fluxes result in morphological changes in the self-assembled state of the block copolymer, which produces a feedback on the conductivity.

Fluxes of matter or energy can lead soft-materials to self-assemble into nonequilibrium structures that differ from those in equilibrium.^12−14^ As an example, electrophoretic fluxes in mixtures of oppositely charged colloids give rise to nonequilibrium forms of organization, such as lanes or bands.^12−14^ These structures are examples of dissipative self-assembly: their existence depends on continuous input and dissipation of energy to maintain the material away from thermodynamic equilibrium.^15^ Moreover, the transition to dissipative structures affects the transport properties of the assembly, leading in the above example to an insulator-conductor transition.^12^

This work introduces a simulation methodology to study PEO–PS+LiTFSI *in-operando* conditions and tens-of-nm length scale. While the PEO–PS+LiTFSI system has been the subject of several theoretical and simulation studies in the past,^16−28^ these prior investigations focused on the material in thermodynamic equilibrium. Predicting its nonequilibrium morphology poses significant challenges because of the disparity of time scales involved in ion and polymer motion and the need to model device-relevant length scales. We address here this problem by combining a generalized-diffusion approach^29,30^ with the Single-Chain-in-Mean-Field (SCMF) algorithm, originally developed for neutral salt-free polymer melts.^31,32^ This combined strategy results is a multiscale theoretical framework, where the polymer dynamics and ions fluxes are self-consistently treated at different levels of approximation. Our simulations successfully reproduce the local morphology changes previously observed in experiments in the presence of ionic currents.^8,9^ We systematically explore the role of polymer-electrode affinity, applied potential, and salt concentration on the morphology and current of the electrolyte. These studies provide a molecular picture of the transport mechanisms operating in PEO–PS+LiTFSI and reveal the emergence of dissipative structures. In particular, when the lamellae are parallel to the electrode, our theory predicts their transformation to a bicontinuous morphology for large applied voltage biases, which maximizes the entropy production and is not anticipated from the equilibrium behavior of the system.

## Theoretical Methods

### Coarse-Grained Modeling of Block Copolymer/Salt Systems

Our model for PEO–PS+LiTFSI assumes that both, the cations and the anions, retain their mobility in the melt,^20,21^ in contrast to other previous approaches^16−19^ that assumed Li^+^ ions to be strongly bound to the ethylene-oxide (EO) groups in PEO. The SCMF theory for block copolymer electrolytes considers a volume *V* with a fixed number of ions *N*_α_ (α = +, – for lithium cations and TFSI^–^ anions, respectively) and a fixed number of polymer chains, *n*_p_. The chains have *N*_PEO_ and *N*_PS_ segments of PEO and PS, respectively, and a total number of segments of *N* = *N*_PEO_ + *N*_PS_. Each of these segments corresponds to multiple chemical monomers, see Molecular Model section. Our theoretical framework approaches the dynamics of PEO–PS+LiTFSI with a multiscale strategy that exploits the dissimilar time scales of ion and polymer motion.

We briefly describe the soft, coarse-grained model and the SCMF algorithm for block copolymer and salt below, with special emphasis on the new contributions that result from the presence of salt and electrostatic interactions. We refer the reader to the Supporting Information (SI) and previous works^31−33^ for additional details.

Let us consider the free energy functional in eq 1, , that models a highly coarse-grained system where the PEO and PS polymer segments are located at positions **r**_*i*,*j*_ (where *i* = 1, ···, *n* and *j* = 1, ···, *N* indicate the chain index and the segment number along a chain, respectively). We decompose  into strong bonded interactions, , along the backbone of the copolymer chains and weak nonbonded interactions, .
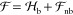
1In eq 1, the bonded interactions, , are modeled as harmonic springs.^31^

The nonbonded interactions, , are expressed as a functional of the average number densities of the ions, ρ_+_ and ρ_–_, the electrostatic potential, ψ, and the normalized densities of the polymer segments,^31^ ϕ_PEO_ and ϕ_PS_, given by eq 2
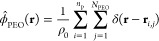
2

The double sum runs over all coarse-grained segments of type PEO, and ρ_0_ = *Nn*_p_/*V* denotes the average polymer-segment number density. The hat indicates that the normalized density explicitly depends on the segment positions. An analog expression holds for ϕ̂_PS_(**r**).

The functional  has different contributions
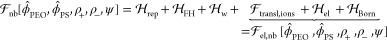
3The first three terms only depend on the segment densities, ϕ̂_PEO_ and ϕ̂_PS_.  represents the repulsive polymer-segment interactions (i.e., excluded volume), which penalize deviations of the local polymer density from its average.
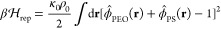
4where β = (*k*_B_*T*)^−1^. As discussed below, we assume that the volume fraction of polymer segments is much larger than that of ions. While the model can be straightforwardly generalized to account for a finite volume of the ions, this would require introducing additional parameters in the theory.

Short-range interactions are given by the Flory–Huggins energy of mixing, 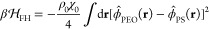
5where χ_0_ is the Flory–Huggins parameter for PEO–PS.

The third term, , accounts for the nonelectrostatic polymer-electrode interactions, which we take as square-well potentials with range σ
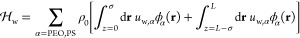
6where *z* denotes the Cartesian coordinate perpendicular to the electrodes. This term allows us to tune the surface of the electrodes to be selective for PEO or PS segments, or to be nonselective (neutral). For further details of the terms , , and  we refer the reader to previous work for salt-free block copolymers.^31−33^

The following three terms in eq 3—, , and —are absent in the soft, coarse-grained model for salt-free polymers.  is the contribution from the translational entropy of the ions
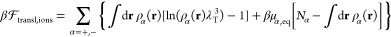
7

In eq 7, λ_T_ is the thermal de-Broglie wavelength, which is without thermodynamic consequences for the following. ρ_α_(**r**) with α = +,– denotes the number density of ion species α. The chemical potentials, μ_α,eq_, of the ions act as Lagrange multipliers to control the number of ions, *N*_α_, in the system. Note that this equation implies that there is a nonzero Li^+^ concentration in the whole system (including in PS-rich regions). Some previous models assumed strong binding of Li^+^ to the PEO backbone (which thus behaves as a polyelectrolyte).^16−19^ In contrast, in our model, as well as in other models in the literature,^20,21^ Li^+^ preferentially locates in the PEO-rich regions due to their higher dielectric constant. These two approaches—strong chemical binding vs solvation by the polymeric solvent—are two different coarse-grained approximations to model the Li^+^-PEO interaction. We choose here the solvation approach as it naturally allows to describe Li^+^ fluxes using a generalized diffusion equation.

Finally, the electrostatic energy of the system is given by eq 8
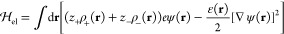
8where *z*_Li^+^_ = +1 and *z*_TFSI^–^_ = −1. In this term, ψ(**r**) and ε(**r**) are the electrostatic potential and dielectric permittivity at position **r**, and *e* denotes the elementary charge. A key aspect of our model is that the dielectric permittivity depends on the local polymer composition, which drives the partition of the salt toward the PEO domains through the Born-energy term (discussed next). More specifically, we use the constitutive relation
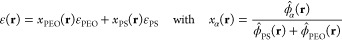
9where ε_*i*_ denotes the dielectric permittivity of the pure polymer α and *x*_α_(**r**) is the local fraction of polymer segments of type α = PEO or PS.

The next term is the Born energy of the ions^20^
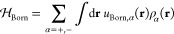
10*u*_Born_,α(**r**) in eq 10 is the Born energy of the ion α at position **r**
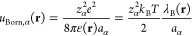
11In eq 11, *a*_α_ denotes the radius of the ion α, and λ_B_(**r**) is the position-dependent Bjerrum length.

It is important to mention at this point that we omitted the contributions of the ion densities to the steric repulsions (eq 4) the nonelectrostatic interactions (eqs 5 and 6), and the local dielectric constant (eq 9). These approximations are justified by the fact that the ions are a minority component of the material (we estimate an ion volume fraction of ∼0.09 for the system under study and a Li^+^:EO ratio, *r* = 0.08) and that the main contribution to salt-induced morphology changes in PEO–PS is the preferential solubility of the ions in the PEO block, which is modeled by . The latter claim is supported by previous theoretical work^18,19^ that properly captured important trends for PEO–PS+LiTFSI in equilibrium despite neglecting the finite volume of the ions. Moreover, the inclusion of the ions in the contributions mentioned above would require parameters that are difficult to independently estimate (i.e., dielectric permittivity of the “pure” ions, Flory–Huggins and ion-surface interaction parameters).

As we mentioned above, we simplify the computational problem by exploiting the separation of the different time scales in the system. First, the electrostatic potential equilibrates instantaneously (compared with molecular motions) and, thus, ψ is always a functional extremum of . The condition,  results in the Poisson equation
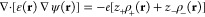
12Thus, ψ[ϕ̂_PEO_, ϕ̂_PS_, ρ_+_, ρ_–_] is not a dynamic degree of freedom.

### Dynamics of Fast Ions and Slow Polymers

The fluxes of the ions are modeled by a generalized diffusion equation.^29,30^ The flux, **J**_α_, of ion species α is given by linear response
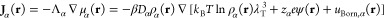
13where *z*_Li^+^_ = +1 and *z*_TFSI^–^_ = −1 and the Onsager coefficient, Λ_α_ = β*D*_α_ρ_α_, is proportional to the self-diffusion coefficient, *D*_α_ of ion species α and its local density. Off-diagonal elements of the Onsager matrix, e.g., coupling gradients in the chemical potential of PEO segments to ion currents are ignored. In the second equality, we obtained the chemical potential of ion α from the free-energy functional, , eq 1, as .^30^Equation 13 is a generalized diffusion equation (model-B dynamics^34^) because it contains additional driving forces beyond the concentration gradient. In this work, these driving forces are the gradients of electrostatic potential and Born energy, which depend on the polymer distribution via the local dielectric permittivity, ε[ϕ̂_PEO_, ϕ̂_PS_].

The following continuity equation relates the ion fluxes and density change
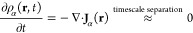
14

To solve our theory, we assume that the ions immediately reach a steady state with respect to the instantaneous polymer configuration, i.e., the ion fluxes **J**_α_(**r**) are divergence-free (transverse). This assumption of time scale separation is of course fulfilled once the simulation reaches the NESS for the polymer degrees of freedom, which is the case for most of the results presented below. On the other hand, its applicability to the transient states that lead to the NESS requires the characteristic diffusional time scale of the polymer chains, τ_R_ = *R*_e_^2^/*D*_p_ (where *D*_p_ and *R*_e_ are the diffusion coefficient and size of a polymer chain) to be much larger than the time scale required for the ions to equilibrate across the electrochemical cell, τ_s_ ≈ *L*^2^/*D*_α_ (where *L* is the thickness of the cell). Note that a local change of the polymer density may result in a global redistribution of the salt ions, whereas the polymer-segment density only varies on the scale of the periodicity, *h* ∼ *R*_e_. Unfortunately, *D*_p_ ≪ *D*_α_ has not been measured for the system under consideration, which precludes a quantitative comparison. Note, however, that assuming that the ions reach a steady state at each polymer configuration is a better approximation for the thin cells considered in the simulation, *L* = 100 nm, τ_s_ ≈ *L*^2^/*D*_α_ = 1 ms, than for the thicker cells used in Li-ion batteries, *L* ∼ 10 μm, τ_s_ = 10 s, or SAXS experiments, *L* ∼ 1 mm, τ_s_ = 27.8 h^10^.

The polymer configurations evolve by a Monte Carlo (MC) simulation using the Single-Chain-in-Mean-Field (SCMF) algorithm.^31,33^ Local random displacements of the polymer segments are proposed, biased by the strong bonded forces.^35^ Whereas the strong bonded forces, , are exactly accounted for, the weak nonbonded interactions are approximated by the quasi-instantaneous fields. The local displacement of a polymer segment, *i*,*j*, from **r**_*i*,*j*_ to **r**_*i*,*j*_^′^ results in a change Δϕ_α_(**r**) = [δ(**r** – **r**_*i*,*j*_^′^) – δ(**r** – **r**_*i*,*j*_)]/ρ_0_ of the normalized density (where α is the type of the polymer segment) and the concomitant change of the nonbonded free-energy,  is approximated by eq 15
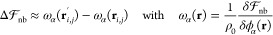
15The different contributions to the external field, ω_α_, are discussed in detail in the SI (eqs S1–S8). Briefly, it depends on the local density of polymer species and the gradient of the electric potential, ψ(**r**), exerting a dielectrophoretic force on the noncharged polymer segments.^36,37^ It also contains a contribution from the Born-energy of the system (which depends on the local ion density), which drives high-dielectric-constant segments to salt-rich regions.

Densities and fields are calculated on a collocation grid, and the quasi-instantaneous fields are updated after every MC step using the instantaneous values of the polymer segment densities, ϕ_α_(**r**), and the stationary values of the ion densities and the electric potential at a given polymer density, according to the self-consistent eqs 12, 13, and 14, respectively.

### Local Electroneutrality Approximation

The solution of the coupled ion diffusion and Poisson eqs (eqs 12, 13, and 14) at every (polymer) MC step is challenging and computationally costly. In low-dielectric materials (such as polymer melts), large deviations from local electroneutrality are energetically prohibitive, thus we invoke the local electroneutrality approximation
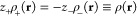
16for all **r** (and thus also *z*_+_*N*_+_ = −*z*_–_*N*_–_ ≡ *N*_charge_). It is very important to note that the use of the local electroneutrality approximation does not imply that the electrostatic potential is constant in the system, but rather that it is obtained from eq 16 instead of from the Poisson equation, eq 12.

Time scale separation, eq 14, and electroneutrality, eq 16, yield
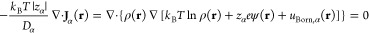
17Adding and subtracting eq 17 for α = +,–, we obtain
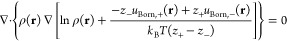
18
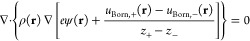
19

Using the equilibrium solution (ρ_eq_, see eq 21 below), we rewrite eq 18 as
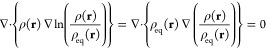
20

In the numerical calculations, we discretize eq 20 and solve it before every (polymer) MC step, using an iterative solver for nonlinear equations.^38^ This procedure is significantly simpler than simultaneously solving the coupled Poisson equation, eq 12, and the generalized diffusion equation, eqs 13 and 14. Additional information on the numerical simulation algorithm and the calculation of the total ionic currents is provided in the SI.

In equilibrium, the condition  simply yields
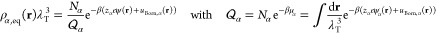
21where  denotes the single-ion partition function. Invoking the local electroneutrality condition, eq 16, we solve for the electrostatic potential
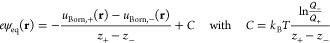
22This result is consistent with eq 19. Inserting it into eq 21, we obtain  with a constant *C*′, consistent with eq 18. Equation 22 reveals that the electric potential profile in the PEO–PS system can be traced back to the difference in the Born energy of the ions, which results from their different ionic radii, eq 11. Equation 22 still requires a self-consistent calculation of  but that calculation is much faster and easier to parallelize than the full solution of the Poisson equation, eq 12.

In the SI we provide an extensive analysis of the validity of the electroneutrality approximation (Figure S1) and show that the electroneutrality approximation introduces a negligible error in the ω_α_(**r**) fields (with α = PEO and PS) and correctly predicts the electrostatic potential and ion densities in those conditions where the block copolymer forms ordered domains.

### Molecular Model

We parameterized our coarse-grained model to approximately match the properties of PS(1.7)PEO(1.4), where the number in parentheses refer to the average block length in units of kg/mol. This material has been extensively studied by Balsara and co-workers, who published its equilibrium phase behavior and morphology changes *in operando* conditions.^8,11^ In our polymer model, a single coarse-grained segment represents more than one chemical repeat unit.^31^ Choosing the coarse-grained segment to have the mass of a single Kuhn segment for PEO, 117 g/mol,^39^ results in mapping each PEO and PS coarse-grained segments onto 2.65 and 1.09 chemical repeat units, respectively. Therefore, there are 12 PEO and 15 PS coarse-grained segments per polymer chain. Using a statistical segment length of 0.77 nm^40,41^ results in an unperturbed chain dimension of *R*_e,0_ ≈ 4 nm. The number density of chains (chosen to agree with the experimental number densities of PEO and PS) was 0.21 chains/nm^3^. The dielectric constants of the polymers were ε_PEO_/ε_0_ = 2.85^42^ and ε_PS_/ε_0_ = 2.6,^43^ with ε_0_ being the vacuum permittivity. The ionic (Born) radii were *a*_Li^+^_ = 0.08 nm^44^ and *a*_TFSI^–^_ = 0.35 nm.^45^ We fixed κ_0_*N* = 100 and used  with *a* = 58.81 and *b* = −0.0602.^46^ For the temperature used in most simulations, *T* = 90 °C, and the number of chemical repeat units in PS(1.7)PEO(1.4), *N* = 48.2, we get χ_0_*N* = 4.9; therefore, PS(1.7)PEO(1.4) is a disordered melt in the absence of added salt. The diffusion coefficients, *D*_α_, of Li^+^ and TFSI^–^ were both fixed to 10^–7^ cm^2^/s.^47^

## Results and Discussion

### Structure of PEO–PS+LiTFSI in Equilibrium

**Figure 1:**
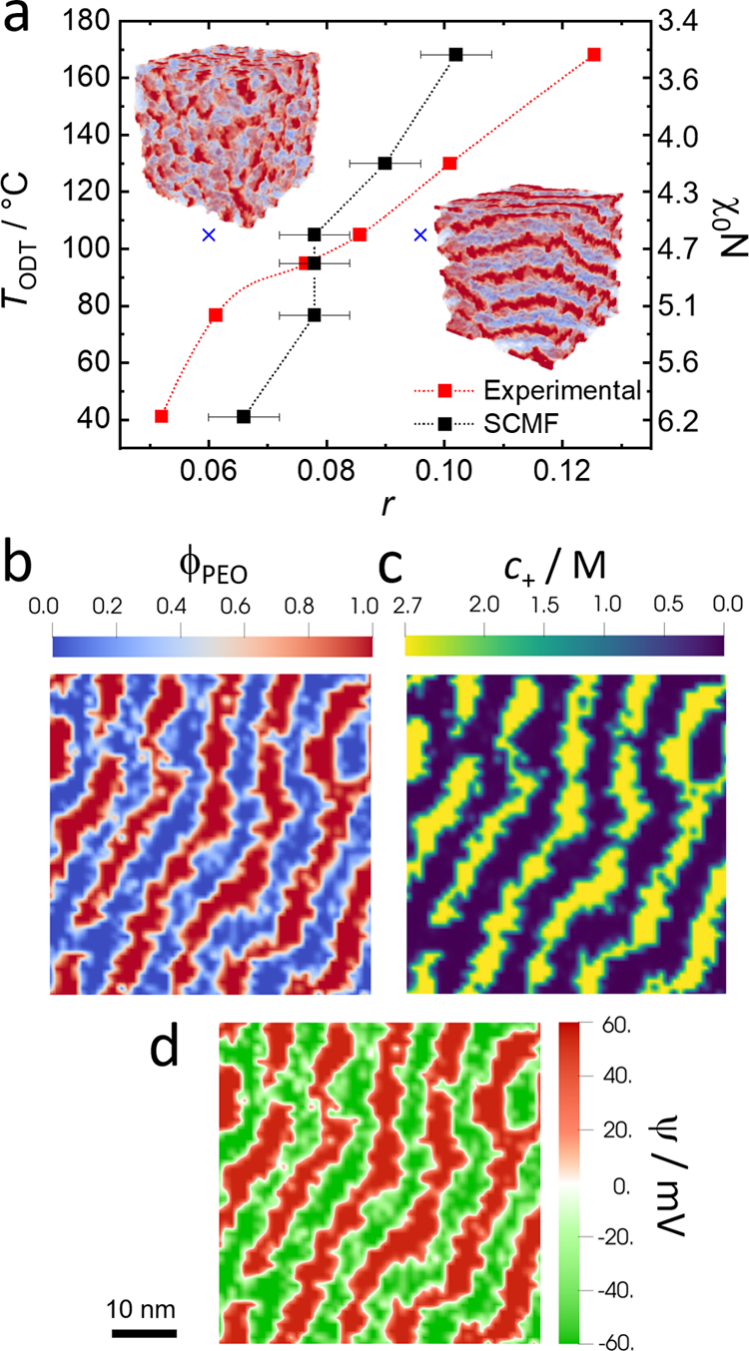
(a) Comparison of the order–disorder-transition (ODT) temperature as a function of the Li^+^:EO ratio, *r*, predicted by simulations (black symbols) and measured in experiments^11^ (red symbols) for the system PS(1.7)PEO(1.4). The insets show the structure of the system in the conditions indicated by crosses. The right ordinate axis shows the values of χ_0_*N* for each temperature. (b–d) Color maps showing the volume fraction of PEO segments (b), the molar concentration of cations (c), and the electrostatic potential (d) for the lamellar phase indicated by a cross in panel a (*T* = 104.9 °C).

Let us first consider a bulk PEO–PS+LiTFSI melt in equilibrium. We estimated the order–disorder transition (ODT) by visual inspection of long (∼800 τ_R_) equilibrium simulations for increasing values of the ratio, *r*, of Li^+^:EO units at fixed T (the errors were estimated from the step of *r*). Figure 1a shows that the ODT temperature increases roughly linearly with *r*, in good agreement with experimental observations from different groups.^6,8,11,48−50^ The *T*_ODT_ vs *r* curve also agrees reasonably well with that measured experimentally by Balsara and co-workers,^8,11^ although the theory overestimates its slope. This discrepancy may result from the approximations in our model, such as not considering anion–cation pairing and Li^+^-EO binding (which would reduce the number of free ions and thus the effect of *r* on *T*_ODT_) or neglecting the ion volumes (which would increase the steric repulsions in the PEO phase, thereby penalizing microphase separation). The domain spacings predicted by our model (7.8 and 8.4 nm for *r* = 0.1 and 0.15, respectively) also increase with *r*, once again in agreement with experimental observations from different groups.^6,11,51,52^ Moreover, they are of the same order of magnitude as the experimental values for the same system and conditions (7.6 and 7.7 nm^11^), although the theory overestimates the swelling of the lamellae by increasing *r*. In this regard, we note that including ion binding/pairing effects would tend to decrease the domain spacing, see Figure 6 in ref (19).

The salt-induced ODT in the PEO–PS melt results from the preferential localization of the ions in the PEO domains. Figure 1b shows the composition of the lamellar phase along a two-dimensional (2D) cut of the system. PEO-rich regions have a higher dielectric constant than PS-rich ones, so the concentration of Li^+^ ions is higher in the PEO domains, cf. Figure 1c. The concentration of TFSI^–^ anions (not shown) exactly matches that of Li^+^ because of the electroneutrality approximation, see Local Electroneutrality Approximation section in the Theoretical Methods section. The Li^+^ cations are smaller than the TFSI^–^ anions, therefore they have a stronger tendency to localize in the high-dielectric domains (see Born energy in eq 11). Hence, the electrostatic potential in the PEO domains is more positive than that in the PS domains to balance the difference in the Born energies of both ions and yield the same local concentrations of Li^+^ and TFSI^–^ (see Figure 1d).

It is important to note that salt-free PS(1.7)PEO(1.4) is disordered at all temperatures,^8,11^ χ_0_*N* ≲ 6.2 at *T* = 40 °C, therefore the ODT transition is not mainly driven by the incompatibility of the blocks, but rather by the enrichment of the salt in the PEO domains. In other words, the ODT reduces the free energy of the system by creating high-dielectric-constant domains and localizing LITFSI in them. As expected, increasing *r* favors the ordered phase, whereas increasing the temperature favors the disordered phase because it lowers the relative weight of the Born energy with respect to the translational entropy of the ions.

### Structure of PEO–PS+LiTFSI *In-Operando* Conditions

**Figure 2:**
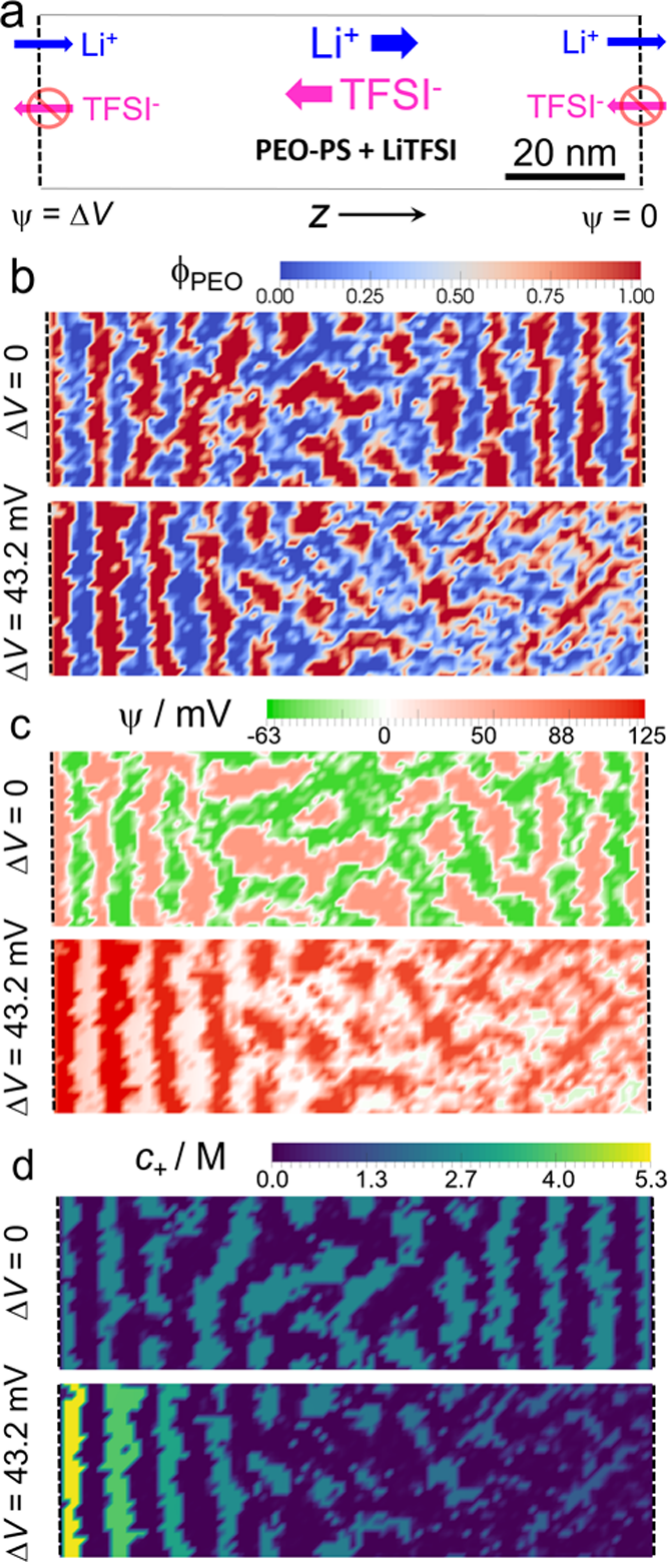
(a) Sketch of the electrochemical cell used for the calculations, showing our convention for the applied electric bias and ionic fluxes. As in an experimental Li^+^ battery, the electrodes consume/generate Li^+^ via electrochemical reactions, but they completely block TFSI^–^ anions. (b–d) Color maps of the local fraction of PEO segments (b), the electrostatic potential (c) and the molar concentration of cations (d) under equilibrium conditions, Δ*V* = 0, and in the nonequilibrium steady state at Δ*V* = 43.2 mV. Calculations were performed at *T* = 90 °C and electrode surfaces that selectively attract the PEO block. The cell thickness is *L* = 100 nm. Other calculation parameters are given in the Molecular Model section.

In the following, we consider ion transport in a thin electrochemical cell, containing PEO–PS+LiTFSI. The cell—sketched in Figure 2a—is aligned with the *z* direction and has a volume of 30 × 30 × 100 nm^3^. Periodic boundary conditions are applied in *x* and *y* directions, and planar electrodes are located at *z* = 0 and *z* = *L* = 100 nm. In the example shown in this section, the electrode surface has a preference for the PEO block, which gives rise to an alignment of lamellae parallel to the electrode (see polymer structure in Figure 2b).

Following the experiments of Balsara and co-workers,^8,9,11^ we model a symmetric cell comprising two lithium metal electrodes, which display a fast and electrochemically reversible kinetics for the production/consumption of Li^+^. Therefore, fluxes of Li^+^ ions from/into the electrodes are allowed. The right electrode is grounded, ψ = 0, and a potential ψ = Δ*V* is applied to the left electrode. Note that in this configuration Li^+^ flows to and is consumed in the negative electrode, as it happens in standard Li-ion and Li-metal batteries during charging. Because of the global electroneutrality requirement, the influx and outflux cancel and the total number of Li^+^ remains unaltered. On the other hand, both electrodes are completely blocking for TFSI^–^, so the flux of this anion at their surfaces is zero. The SI provides a detailed expression for the boundary conditions, including the calculation of the electric current density through the cell, *j*, generated by the Li^+^ fluxes at the electrodes.

As discussed in the introduction, in the steady state the LiTFSI concentration increases near the positive electrode and decreases near the negative electrode. In these conditions, there is a diffusive TFSI^–^ flux toward the negative electrode that exactly cancels the migration flux of this anion toward the positive electrode created by the applied electric bias. The concentration of Li^+^ follows that of TFSI^–^ to maintain local electroneutrality. Starting from an equilibrium lamellar morphology (top panel in Figure 2b), the decrease in salt concentration near the right electrode triggers a local transition to the disordered phase, in agreement with the experimental observations of Balsara and co-workers.^8,11^Figure 2c, 2d show the local electrostatic potential and the Li^+^ molar concentration (*c*_+_/*M* = ρ_+_dm^3^/*N*_A_, where *N*_A_ is Avogadro’s number) in the cell, both for equilibrium, Δ*V* = 0, and nonequilibrium steady-state conditions. In the latter case, the ψ and *c*_+_ profiles result from a combination of the local heterogeneities produced by the lamellar morphology and the average gradient at the cell level generated by the applied potential bias. Note, for example, that the salt concentration is homogeneous within each lamella, but this concentration is smaller for the lamellae far from the left electrode than for the ones close to it.

### Effect of the Li^+^:EO Ratio, *r*, on the Steady-State Morphology

**Figure 3:**
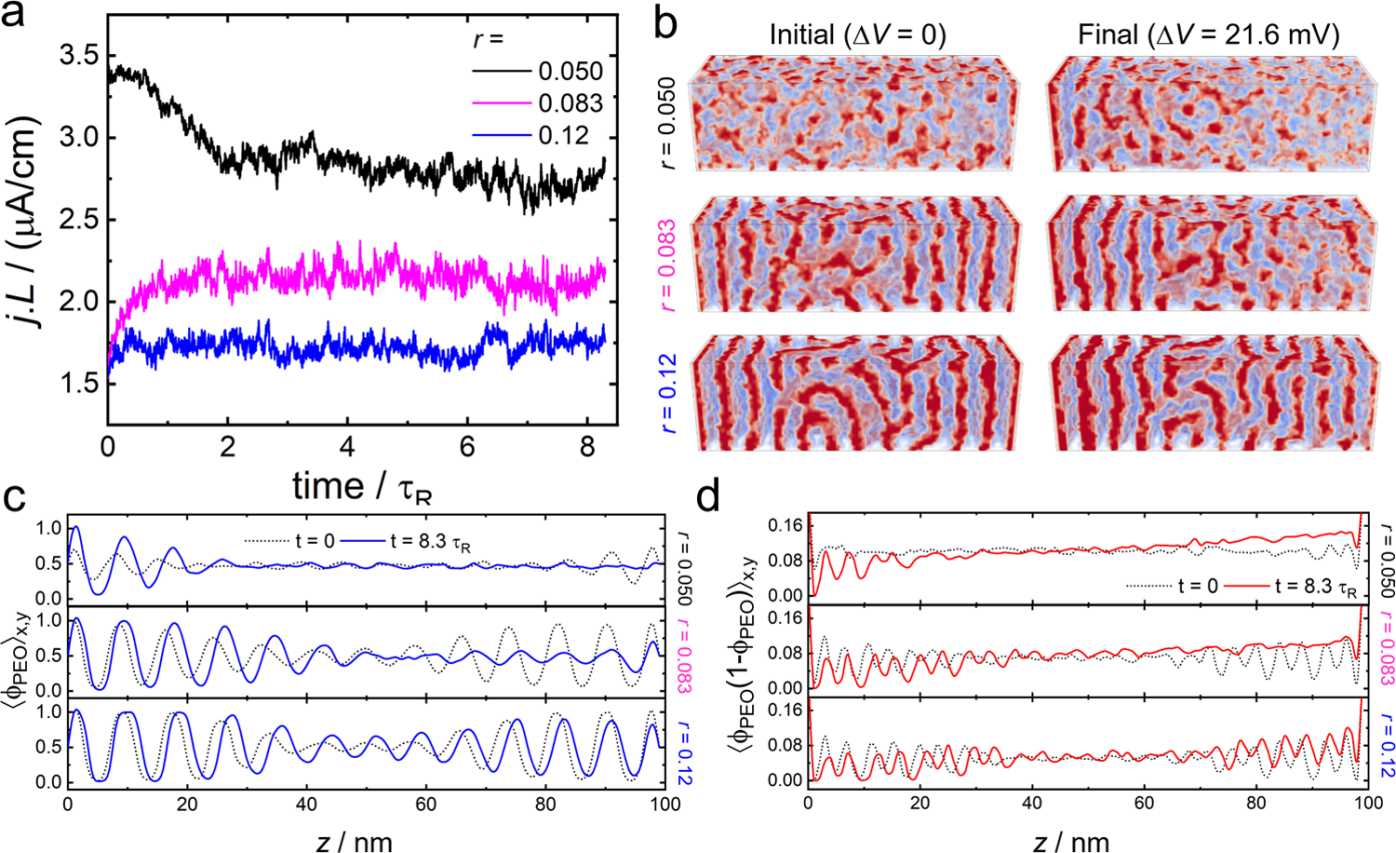
(a) Predicted current-length product, *jL*, vs simulation time for different values of *r* = Li^+^:EO. In all cases, the simulations were started from the equilibrium state, Δ*V* = 0, and a bias of Δ*V* = 21.6 mV was applied at *t* = 0. (b) Snapshots of the initial and final states of the simulation. PEO and PS blocks are shown in red and blue, respectively. The cell thickness is *L* = 100 nm. (c, d) Plots of the *x*,*y*-averaged molar fraction of PEO (c) and the order parameter ⟨ϕ_PEO_(1 – ϕ_PEO_)⟩_*x*,*y*_ (d) *vs z* for the three systems shown in panels a and b. Other calculation parameters as in Figure 2.

We now focus on the effect of the Li^+^:EO ratio, *r*, on the current and morphology of the system. Starting from a system equilibrated at Δ*V* = 0, we applied a bias of Δ*V* = 21.6 mV at *t* = 0 and calculated the time evolution of the current density, *j*, from the simulations, see Figure 3a. The time in this figure is expressed in terms of the relaxation time scale of the polymer, τ_R_ = *R*_e_^2^/*D*_p_. Note also that we plot *jL*, rather than just *j*, because, as discussed in refs (9,11), *jL* allows to compare cells with different thicknesses.

The current transients in Figure 3a last approximately 2τ_R_, which is the time required for the systems to evolve from their initial state, prepared at Δ*V* = 0, to their final nonequilibrium steady states (NESS) at Δ*V* = 21.6 mV. For large applied biases, we observed longer equilibration times, as discussed below. The system with *r* = 0.05 is initially disordered (see Figure 3b), and it becomes ordered near the left electrode in the NESS due to the local increase in salt concentration. The formation of lamellae parallel to the electrode, therefore, leads to a decay in the current density (black line in Figure 3a). The mechanism of ion transport through lamellae that are parallel to the electrode is discussed in a following section. The system with *r* = 0.083 exhibits a lamellar morphology, although it is very close to the ODT. In its NESS, the region next to the right electrode becomes depleted of salt and, thus, it undergoes a transition to the disordered phase. This transition is accompanied by an increase of the current (magenta line in Figure 3a) because the disordered phase has a smaller resistance than the lamellae parallel to the electrode. Finally, for *r* = 0.12, the system forms a stable lamellar phase that retains its morphology throughout the entire system in nonequilibrium conditions. Thus, the current remains approximately constant during the simulation (blue line in Figure 3a).

Figure 3c plots a profile of the normalized PEO density, ⟨ϕ_PEO_⟩_*x*,*y*_, averaged over the *x*,*y* plane *vs z* for the structures shown in Figure 3b. The figure shows that for *r* = 0.12 the lamellar morphology is maintained in the NESS, however the distances between lamellae increase near the positive electrode, *z* = 0, and decrease near negative electrode, *z* = *L*, which agrees with the SAXS experiments of Galluzo et al.^9,11^ Local swelling/deswelling of the lamellae results from the effect of salt concentration on the domain spacing. It is interesting to note that our model does not consider the finite volume of the ions in the repulsive contribution to the free energy (see compressibility term, eq 4), thus swelling is mainly produced by the increase of the ion pressure inside the PEO domains due to the increase of the local salt concentration. This argument is supported by a quantitative analysis of the profiles in Figure 3c, which show that the average swelling, Δ*h*, of the PEO layers in the left half of the cell, Δ*h* ≈ 0.34 nm, is larger than that of the PS layers, Δ*h* ≈ 0.08 nm. As expected, the PEO layers in the right half of the system contract more, Δ*h* ≈ −0.22 nm, than the PS ones, Δ*h* ≈ −0.003 nm (the initial equilibrium thickness for both PEO and PS layers are 4.1 ± 0.1 nm).

Figure 3d plots the laterally averaged order parameter ⟨ϕ_PEO_(1 – ϕ_PEO_)⟩_*x*,*y*_*vs z*. This order parameter reaches a maximum at ϕ_PEO_ = 0.5, (close to the average volume fraction of the disordered phase, ϕ̅_PEO_ = 12/27 = 0.44), and it is minimal inside the segregated domains. The figure reveals that the profile is leveled for Δ*V* = 0, and becomes tilted in the NESS, Δ*V* > 0, which indicates its usefulness to detect position-dependent changes in local order, even in cases where they are not easily detectable by visual inspection (e.g., *r* = 0.12).

### Coupling between Current and Morphology

**Figure 4:**
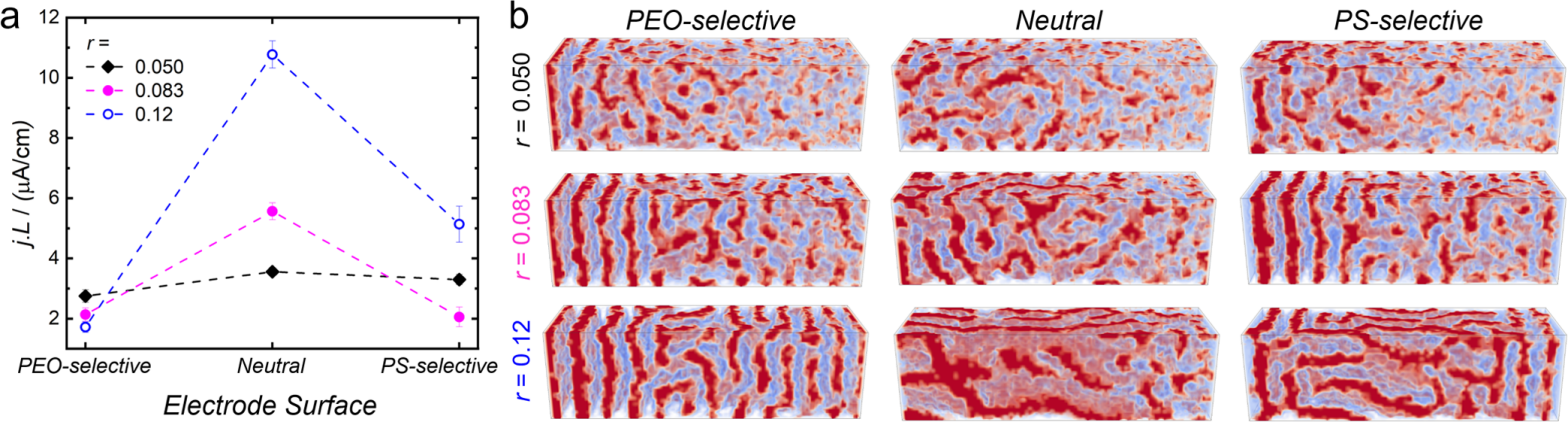
(a) Steady-state current-length product, *jL*, at an applied bias of Δ*V* = 21.6 mV for different *r* and types of electrode surface. (b) Snapshots of the systems in the NESS. PEO and PS blocks are shown in red and blue, respectively. The simulation time was 8.3 τ_R_. The cell thickness is *L* = 100 nm. Other calculation parameters as in Figure 2.

The results in the previous section demonstrate a coupling between ion-transport and polymer morphology in PEO–PS+LiTFSI. In Figure 4, we analyze in detail the effect of *r* and the selectivity of the electrode surface on the current and morphology of the system. In these calculations, we tuned the selectivity of the electrode surface for the PEO or PS blocks (or none of them, i.e., a neutral surface) by varying the surface-polymer attraction strength (see eq 6). It is worth mentioning that the electrode surfaces are selective for PEO for *u*_s,PEO_ = *u*_s,PS_ = 0 (results shown so far). We ascribe this behavior to the higher ionic pressure in the PEO domains than in the PS ones. Hence, PEO domains have a slightly smaller polymer density than PS domains and, thus, localizing them near a hard surface results in a smaller entropic penalty from the loss of allowed polymer conformations.^53^ The asymmetry of the diblock—the polymers have 12 PEO and 15 PS coarse-grained beads—also contributes to the surface preference of the shorter PEO block because chain ends are enriched at the sharp density gradient of the surface.^54^ Moreover, in the presence of a voltage bias, Δ*V* > 0, the salt concentration is higher at the electrodes, favoring the enrichment of the component with the higher dielectric constant, PEO.^55^ The selectivity of the surface toward the PEO block can be offset by introducing a repulsive interaction between the surface and PEO beads to obtain neutral (*u*_s,PEO_ = 5 *k*_B_*T*) or PS-selective (*u*_s,PEO_ = 10 *k*_B_*T*) surfaces.

For a PEO-selective surface, already discussed in Figures 2 and 3, the current decreases for increasing *r* (see Figure 4a). This is ascribed to the formation of multiple lamellae parallel to the electrodes for large *r*, which increase the cell resistance. The cell with neutral surfaces displays much higher ion currents than the cells with PEO- and PS-selective electrodes because the lamellae are no longer parallel to the electrodes (cf. Figure 4b). Unlike the PEO-selective case, the current increases with increasing *r*, for the neutral surface, which is simply explained by the increase in the number of charge carriers, Li^+^ and TFSI^–^, with *r*. For the PS-selective electrodes the current nonmonotonically depends on *r*, likely because of the combination of the two opposing effects—polymer morphology and total number of charge carriers—discussed above.

### Effect of Applied Bias on the Current and Structure of the System

**Figure 5:**
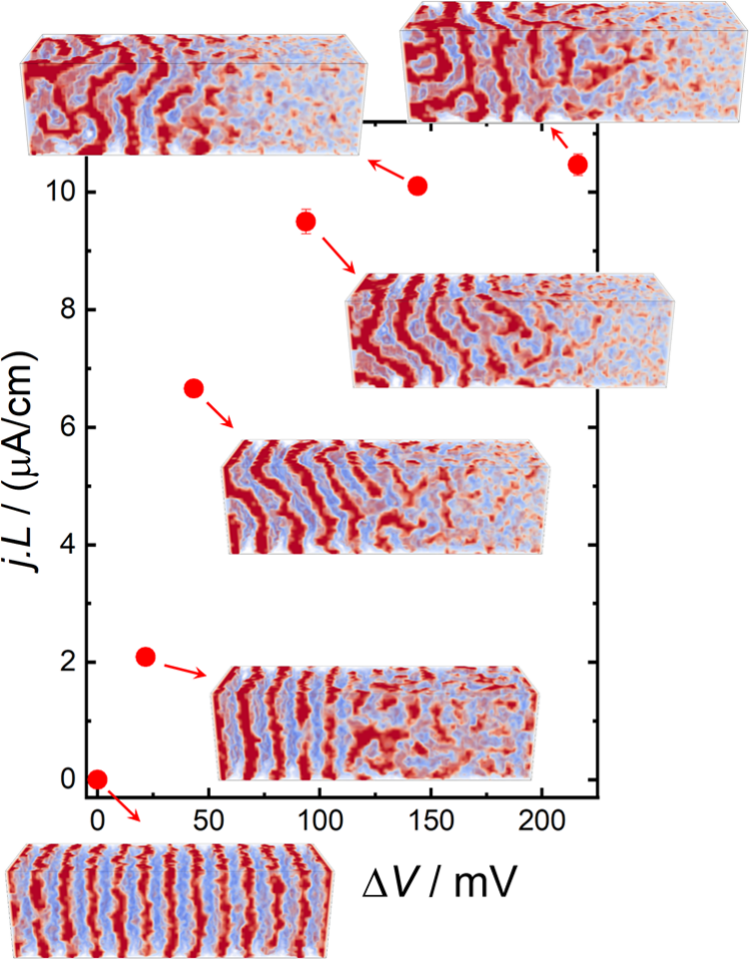
Steady-state current-length product, *jL*, as a function of the electric bias applied between the electrodes, Δ*V*. The insets show snapshots of the system. Calculations for *r* = 0.083 and a PEO-selective surface. The error bars were calculated from the stochastic fluctuations of the current in the simulation (see Figure 3). The simulations were started from a system equilibrated at Δ*V* = 0 and evolved for 91.2 τ_R_. Other calculation parameters as in Figure 2.

Figure 5 shows the current density vs applied potential for a system with *r* = 0.083 and a PEO-selective surface. These calculations were performed for long simulations times (91.2 τ_R_) because we observed slow structural rearrangements for large applied bias. Note that for the applied bias of 21.6 mV used in the previous section, the structure of the system reaches the NESS much faster, on a time scale of a few τ_R_. Figure 5 shows that for an applied bias of Δ*V* ≈ 200 mV, the current density converges to a limiting value of *j*_lim_*L* ≈ 10.5 μA/cm. The value of Δ*V* ≈ 200 mV corresponds to a ratio of cation concentrations on the electrode surfaces of *c*_+_^left^/*c*_+_^right^ = ρ_+_^left^/ρ_+_^right^ ≈ 600 (see eq S12 in the SI), where “left” and “right” indicate the surfaces of the respective electrodes. This result suggests that the limiting current results from the total depletion of the Li^+^ ions near the right electrode, which is the same mechanism operating in homopolymer electrolytes.^56^ It has been shown^11,57^ that the product *j*_lim_*L* is independent of the cell thickness, *L*, so it serves to compare cells of different thicknesses. The value *j*_lim_*L* ∼ 10.5 μA/cm predicted for a simulation cell with *L* = 100 nm is within the range of 6–23 μA/cm experimentally determined for PEO–PS+LiTFSI for the same *r*, but a much thicker cell, *L* = 20 μm. This comparison demonstrates that our results are relevant to understanding the mechanisms operating in Li^+^ batteries.

**Figure 6:**
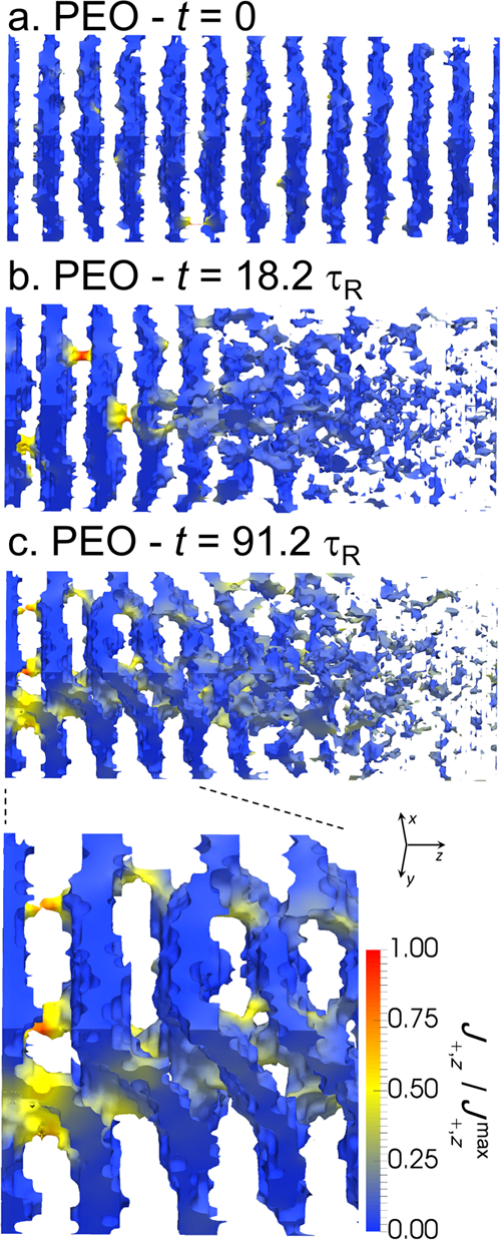
(a–c) 3D image of the PEO component at different simulation times. Regions with ϕ_PEO_ < 0.95 are transparent. The surface is colored according to the *z* component of the flux of Li^+^ cations, *J*_+,*z*_. The calculations correspond to *r* = 0.083, a PEO-selective surface, and Δ*V* = 43.2 mV (*c*_+_^left^/*c*_+_^right^ = 4). Other calculation parameters as in Figure 2.

The snapshots in Figure 5 reveal that increasing the applied bias beyond Δ*V* ≈ 40 mV results in a loss of lamellar order in the left half of the cell. Figure 6 shows the morphology of the system at different times after applying a potential bias of Δ*V* = 43.2 mV to a system initially in equilibrium (Figure 6a). In these plots, the PS blocks (i.e., regions with ϕ_PEO_ < 0.95) have been hidden and the color scale indicates the *z* component of the Li^+^ flux. After applying the potential jump, the right half of the cell becomes disordered in a time scale smaller than τ_R_ due to the depletion of salt, as explained above. In the following 10–20 τ_R_, we observed the formation of PEO channels, piercing the PS lamellae and conducting the Li^+^ ions through them (Figure 6b). As time evolves, the lamellar order is partially lost, and a bicontinuous structure forms (see Figure 6c and Movie S1 in the SI). The formation of this structure is reversible and requires continuous input of energy in the form of an ionic current flowing through the system: if the applied potential is turned off, Δ*V* = 0, the system relaxes back to the lamellar morphology (Movie S2). Interestingly, the loss of lamellar order and the formation of PEO channels through the PS layers does not occur for Δ*V* = 21.6 mV (see Figure 5). Note Li^+^ ions can migrate through the PS domains parallel to the electrode because there is a nonzero Li^+^ concentration in the PS phase.

The formation of the bicontinuous phase cannot be explained by considering the local increase in salt concentration and the equilibrium behavior of the system. For an approximately linear salt concentration profile, the concentration near the left electrode is approximately 2*r*.^8,9^ In equilibrium, we observed that increasing the average salt concentration up to *r* = 0.45 still produces a well-ordered lamellar phase; therefore, the loss of lamellar order observed in Figure 6c (calculation for *r* = 0.083) cannot be explained by the local increase in salt concentration. It is worthwhile to mention that the experimental phase diagram of this system displays a lamella-to-gyroid transition for *r* > 0.18.,^8,11^ which is ascribed to an increase in the effective volume fraction of the PEO domains. This effect is not captured by our model probably because the salt ions have no finite volume.

The formation of the bicontinuous structure may also result from the competition between the selectivity of the surface for PEO (which favors lamellae parallel to that surface) and a mechanism stabilizing lamellae perpendicular to the electrodes. For example, the dielectrophoretic alignment of salt-free block copolymers^36,37,58−61^ tends to orient the internal domain interfaces along the electric field, i.e., perpendicular to the electrode surfaces. The dielectrophoretic force scales as , and the concomitant energy is represented by the ω_α,el_(**r**) -contribution to the total ω_α_ field acting on polymer segments (eq S6 in the SI). We found that setting this contribution to zero does not prevent the formation of the bicontinuous phase and, therefore, this phenomenon cannot be ascribed to the dielectrophoretic force (see Figure S2 in the SI). Other works^62−65^ have considered block copolymers where one microphase contains mobile counterions. In this case, the charge separation due to the response of the ions to the applied electric field may favor the perpendicular orientation. This mechanism cannot explain our results because the local electroneutrality approximation in our model prevents the spatial separation of positive and negative charges.

While most of the mechanisms discussed in the previous paragraph operate in equilibrium systems, the formation of the bicontinuous phase in the PEO–PS+LiTFSI electrolyte occurs in a NESS. The principle of maximum entropy production (MaxEP)^66,67^ dictates that the most stable NESS is the one that maximizes entropy production subjected to the constraints, such as constant applied potential. This principle is expected to apply even far from equilibrium,^66^ i.e., in the nonlinear regime at large applied potential shown in Figure 5. In the present case, entropy is produced by heat dissipation, (67) (where *A* is the lateral area of the system). As expected, the formation of the bicontinuous structure increases the current flowing through the cell (see Figure S3) and, therefore, increases *Ṡ*, in agreement with the MaxEP principle. In summary, the bicontinuous phase is a dissipative structure that cannot be explained in terms of the equilibrium behavior of the system and that forms at sufficiently large Δ*V* in order to maximize entropy production.

## Conclusions

This work introduced a simulation methodology to address the self-assembly of block copolymer electrolytes *in-operando* conditions, *i.e.*, in the presence of ionic currents. Our multiscale approach describes ion fluxes with generalized diffusion equations formulated within a soft, coarse-grained model for polymer motions, thereby leveraging the natural separation of time scales of both phenomena. In this way, ion and polymer motion are treated at different levels of approximation, but within a single, self-consistent theoretical framework.

We applied our simulation methodology to the specific case of PEO–PS+LiTFSI, a material that was the subject of intensive experimental characterizations, both in equilibrium and *in-operando* conditions.^8,9,11^ A good level of agreement was observed between theory and experiment for the equilibrium phase behavior, current-driven morphological changes, and the limiting current-length product. We demonstrated that the configuration where the lamellae are normal the electrode surface is optimal for transport. However, obtaining this orientation requires a nonselective surface and, therefore, a very delicate balance of interactions that can be difficult to obtain experimentally. Arguably, the formation of lamellae parallel to the electrodes is probably the most common experimental situation. We demonstrated that in this configuration, ion transport at high applied biases is enabled by the formation of a bicontinuous phase. This structure cannot be explained by equilibrium mechanisms, such as dielectrophoretic forces. Therefore, we argue that this bicontinuous structure is a dissipative structure that maximizes the current—and thus energy dissipation—in agreement with the principle of maximum entropy production.

Our simulation successfully predicts the disorder-to-lamella and lamella-to-disorder transitions induced by ionic currents that were previously observed for PEO(1.4)–PS(1.7).^8,10,11,47^ For less symmetric block copolymers, Balsara’s group also observed lamella-to-gyroid and hexagonal-to-gyroid transitions, while the gyroid-to-hexagonal transition was not found under the expected conditions. Theoretical investigations of other PEO–PS block copolymer compositions could provide valuable insights into this unexplained observation, offering a deeper understanding of which morphologies are accessible under nonequilibrium conditions. The prediction of these morphologies within our theoretical framework, as well as improving the quantitative comparison with the experiments, will require further refinements of the model, such as explicitly taking into account the volume of the ions. Another unexplained experimental observation is that lamellae swell differently depending on their relative orientation with respect to the ionic current.^9^ While our simulations predict the current-induced swelling of the PEO lamellae, the simulated system is too small to study orientation effects. Further improvements in the efficiency of our methodology (*e.g.*, by developing a GPU implementation of the calculation of ionic fluxes) may grant access to larger systems and address this issue.

A salient feature of our model is its ability to capture nonequilibrium self-assembly. We showed that current and morphology are inextricably coupled in the PEO–PS+LiTFSI system. This coupling results in the formation of gradient structures, which has been experimentally observed,^8,10,11,47^ and dissipative structures, whose existence is neither anticipated from nor explained by the equilibrium behavior of the system. In the future, we plan to explore the presence of dissipative structures in other families of block copolymer electrolytes,^68^ as well as in electronically conducting^69^ and redox-active block copolymers.^70^

## Supporting Information Available

Model and simulation technique, the accuracy of the electroneutrality assumption, the formation of a bicontinuous phase in the absence of dielectrophoretic forces and the current transient for the formation of the bicontinuous phase (PDF)
Simulation trajectories of the formation of the bicontinuous phase is compiled (Movie S1) (MP4)
Simulation trajectories of the decay of the bicontinuous phase is compiled (Movie S2) (MP4)
