# Supplementary material for: Morphology–Transport Coupling and Dissipative Structures in PEO–PS+LiTFSI Electrolytes In-Operando Conditions
Source: ACS Appl Mater Interfaces. 2025 Jan 30;17(6):9278–88. doi: 10.1021/acsami.4c18838 (PMC11826895; doi:10.1021/acsami.4c18838)
Supplement: Supplementary file 1 — am4c18838_si_001.pdf [file am4c18838_si_001.pdf]

Supporting Information
Morphology-transport coupling and dissipative structures in PEO-PS+LiTFSI electrolytes in-operando conditions

Mario Tagliazucchi*,†,‡ and Marcus Müller*,¶

†*Departamento de Química Inorgánica Analítica y Química Física, Ciudad Universitaria, Facultad de Ciencias Exactas y Naturales, Universidad de Buenos Aires, Pabellón 2 C1428EGA, Buenos Aires, Argentina*

‡*Instituto de Química de los Materiales, Ambiente y Energía (INQUIMAE). Ciudad Universitaria, CONICET, Universidad de Buenos Aires, Facultad de Ciencias Exactas y Naturales, Pabellón 2 C1428EGA, Buenos Aires, Argentina*

¶*Institute for Theoretical Physics, Georg-August University of Göttingen, 37077 Göttingen, Germany*

E-mail: mario@qi.fcen.uba.ar; mmueller@theorie.physik.uni-goettingen.de

Coarse-grained modeling of block-copolymer/salt systems

Fields acting on the polymer segments

The fields

$$\omega_\alpha(\mathbf{r}) = \frac{1}{\rho_0}\frac{\delta \mathcal{F}_{\rm nb}}{\delta \hat{\phi}_\alpha(\mathbf{r})} \qquad \text{with } \alpha = \text{PEO or PS} \tag{S1}$$

represent the quasi-instantaneous interaction of a polymer segment with its environment. The different contributions to $\mathcal{F}_{\rm nb}$ in Equation 3 yield

$$\omega_{\rm PEO}(\mathbf{r}) = \omega_{\rm PEO,rep} + \omega_{\rm PEO,FH} + \omega_{\rm PEO,w} + \omega_{\rm PEO,el} + \omega_{\rm PEO,Born} \qquad \text{with} \tag{S2}$$

$$\omega_{\rm PEO,rep}(\mathbf{r}) = \frac{1}{\rho_0}\frac{\delta \mathcal{F}_{\rm rep}}{\delta \hat{\phi}_{\rm PEO}(\mathbf{r})} = k_{\rm B}T\kappa_0\left[\hat{\phi}_{\rm PEO}(\mathbf{r}) + \hat{\phi}_{\rm PS}(\mathbf{r}) - 1\right] \tag{S3}$$

$$\omega_{\rm PEO,FH}(\mathbf{r}) = -k_{\rm B}T\frac{\chi_0}{2}\left[\hat{\phi}_{\rm PEO}(\mathbf{r}) - \hat{\phi}_{\rm PS}(\mathbf{r})\right] \tag{S4}$$

$$\omega_{\rm PEO,w}(\mathbf{r}) = -u_{\rm w,PEO}\left[\Theta(\sigma - z) + \Theta(z - L + \sigma)\right] \text{ with Heaviside function } \Theta \tag{S5}$$

$$\omega_{\rm PEO,el}(\mathbf{r}) = \int {\rm d}\mathbf{r}'\, \underbrace{\frac{\delta \mathcal{H}_{\rm el}}{\delta \psi(\mathbf{r}')}}_{=0} \frac{\delta \psi(\mathbf{r}')}{\delta \hat{\phi}_{\rm PEO}(\mathbf{r})} - \frac{|\nabla \psi(\mathbf{r})|^2}{2\rho_0}\frac{{\rm d}\varepsilon(\mathbf{r})}{{\rm d}\hat{\phi}_{\rm PEO}(\mathbf{r})} \tag{S6}$$

$$\omega_{\rm PEO,Born}(\mathbf{r}) = -\sum_{\alpha=+,-}\frac{e^2\rho_\alpha(\mathbf{r})}{8\pi\varepsilon^2(\mathbf{r})a_\alpha\rho_0}\frac{{\rm d}\varepsilon(\mathbf{r})}{{\rm d}\hat{\phi}_{\rm PEO}(\mathbf{r})} = -\frac{{\rm d}\ln\varepsilon(\mathbf{r})}{\rho_0\,{\rm d}\hat{\phi}_{\rm PEO}(\mathbf{r})}\sum_{\alpha=+,-} u_{{\rm Born},\alpha}(\mathbf{r})\rho_\alpha(\mathbf{r}) \tag{S7}$$

Equation S6 gives rise to a dielectrophoretic force on the noncharged polymer segments.[1,2] The contribution in Equation S7 drives high-dielectric-constant segments to salt-rich regions. Both electrostatic contributions stem from the dependence of the dielectric constant on the local fraction of the different polymer species. *

Simple argument for the equilibrium phase diagram

In equilibrium, we obtain simple expressions for the ion densities and the electrostatic potential. As a rough approximation and to better understand the equilibrium behavior, we can assume that salt ions are completely localized in the high-ε PEO domains and obtain an effective χ parameter for the Born contribution[3,4] ($z_+ = -z_- = 1$, and assuming strict

*If the ion degrees of freedom were *not independent* dynamic degrees of freedom but, instead, were instantaneously related (slaved) to the polymer-segment coordinates (like the electrostatic potential ψ), all terms in $\mathcal{F}_{\rm nb}$ could be expressed in terms of $\hat{\phi}_{\rm PEO}(\mathbf{r})$ and $\hat{\phi}_{\rm PS}(\mathbf{r})$. In this case, the field acting on a PEO segments would pick up an additional term $\Delta\omega_{\rm PEO}(\mathbf{r}) = \sum_{\alpha=+,-} \frac{1}{\rho_0}\int {\rm d}\mathbf{r}'\ \frac{\delta\mathcal{F}_{\rm nb}}{\delta\rho_\alpha(\mathbf{r}')}\frac{\delta\rho_\alpha(\mathbf{r}')}{\delta\hat{\phi}_{\rm PEO}(\mathbf{r})} = \sum_{\alpha=+,-}\frac{1}{\rho_0}\int {\rm d}\mathbf{r}'\ [\mu_\alpha(\mathbf{r}') - \mu_{\alpha,{\rm eq}}]\frac{\delta\rho_\alpha(\mathbf{r}')}{\delta\hat{\phi}_{\rm PEO}(\mathbf{r})}$. In equilibrium, this contribution vanishes, of course.

If we assume translational invariance the term $\frac{\delta\rho_\alpha(\mathbf{r}')}{\delta\hat{\phi}_{\rm PEO}(\mathbf{r})}$ will only depend on the relative distance $\mathbf{r}-\mathbf{r}'$, and we can rewrite the force contribution in the form:

$$
\begin{aligned}
-\nabla\Delta\omega_{\rm PEO}(\mathbf{r}) &= -\sum_{\alpha=+,-}\frac{1}{\rho_0}\int {\rm d}\mathbf{r}'\ [\mu_\alpha(\mathbf{r}') - \mu_{\alpha,{\rm eq}}]\,\nabla\frac{\delta\rho_\alpha(\mathbf{r}')}{\delta\hat{\phi}_{\rm PEO}(\mathbf{r})} \\
&= \sum_{\alpha=+,-}\frac{1}{\rho_0}\int {\rm d}\mathbf{r}'\ [\mu_\alpha(\mathbf{r}') - \mu_{\alpha,{\rm eq}}]\,\nabla'\frac{\delta\rho_\alpha(\mathbf{r}')}{\delta\hat{\phi}_{\rm PEO}(\mathbf{r})} \\
&= -\sum_{\alpha=+,-}\frac{1}{\rho_0}\int {\rm d}\mathbf{r}'\ [\nabla'\mu_\alpha(\mathbf{r}')]\,\frac{\delta\rho_\alpha(\mathbf{r}')}{\delta\hat{\phi}_{\rm PEO}(\mathbf{r})} + \text{boundary terms} \\
&= \sum_{\alpha=+,-}\frac{1}{\rho_0\beta D_\alpha}\int {\rm d}\mathbf{r}'\ \frac{\mathbf{J}_\alpha(\mathbf{r}')}{\rho_\alpha(\mathbf{r}')}\frac{\delta\rho_\alpha(\mathbf{r}')}{\delta\hat{\phi}_{\rm PEO}(\mathbf{r})} + \text{boundary terms} \\
&= \sum_{\alpha=+,-}\int {\rm d}\mathbf{r}'\ \zeta_\alpha\mathbf{v}_\alpha(\mathbf{r}')\frac{\delta\rho_\alpha(\mathbf{r}')}{\rho_0\delta\hat{\phi}_{\rm PEO}(\mathbf{r})} + \text{boundary terms}
\end{aligned}
$$

where $\zeta_\alpha = k_{\rm B}T/D_\alpha$ is the friction coefficient of the ion species α and $\mathbf{v}_\alpha(\mathbf{r}) = \frac{\mathbf{J}_\alpha(\mathbf{r}')}{\rho_\alpha(\mathbf{r}')}$ is the velocity of ion species α at position $\mathbf{r}'$, respectively. This contribution resembles a nonlocal drag force that the nonequilibrium ion current exerts onto the polymer. We assume that the ions relax quasi-instantaneously, *i.e.*, $D_\alpha \to \infty$, and thus $\zeta_\alpha \to 0$. We expect that microphase separation and the presence of the walls will not qualitatively alter this argument. The neglect of the term is also consistent with the vanishing volume of the ions.

incompressibility, $\hat{\phi}_{\rm PEO} + \hat{\phi}_{\rm PS} = 1$),

$$\frac{\chi_{\rm Born}^{\rm eff}}{k_{\rm B}T} \approx r \frac{\varepsilon_{\rm PEO} - \varepsilon_{\rm PS}}{\varepsilon_{\rm PEO}} \frac{\lambda_{\rm B,PEO}}{4} \left(\frac{1}{a_+} + \frac{1}{a_-} \right) \tag{S8}$$

Here $r = \frac{N_+}{f_{\rm PEO}\rho_0 V}$ denotes the ratio of Li^+ and EO units and $\lambda_{\rm B,PEO} \approx 4.0$ nm is the Bjerrum length in PEO at 90ºC. Using the model parameters, we obtain $\frac{\chi_{\rm Born}^{\rm eff}}{k_{\rm B}T} \approx 1.35r$ or $\chi_{\rm Born}^{\rm eff} N \approx 36.5r$. The order of magnitude of this estimate agrees rather well with the slope $\frac{\Delta\chi N}{\Delta r}$ of the ODT transition in Figure 1a, although slighly underestimates its value ($\frac{\chi_{\rm Born,\ simulation}^{\rm eff}}{k_{\rm B}T} \approx 2.8r$).

Validation of the local electroneutrality approximation

We validated the electroneutrality (EN) approximation by comparing results obtained with this approximation with the nonapproximate solution, obtained by solving the Poisson equation for the equilibrium case, $\Delta V = 0$. Figure S1a, b and c show color maps of the fraction of PEO monomers, the electrostatic potential, ψ, and the molar concentration of Li^+ ions for the non-approximated solution. Panel d shows the difference between the concentrations of Li^+ and $TFSI^-$. This difference is zero by definition in the EN approximation. Figure S1d indicates that local electroneutrality is a good approximation inside the domains, but it worsens at their interfaces. This is expected, because the difference of electrostatic potential between the microphase-separated domains generates an electric double layer at their interfaces,[5] where local electroneutrality does not hold. Note that on the PEO side of the interface, the concentration of Li^+ is higher than that of $TFSI^-$, whereas the opposite occurs on the PS side. The reason for this distribution is that the Li^+ ion is smaller than the $TFSI^-$ ion and, therefore, it has a higher affinity for the block with the higher dielectric constant, PEO.

Figure S1e shows the distribution of the error in the cation concentration, resulting from applying the EN approximation. Not surprisingly, the error is mainly located at the interfaces

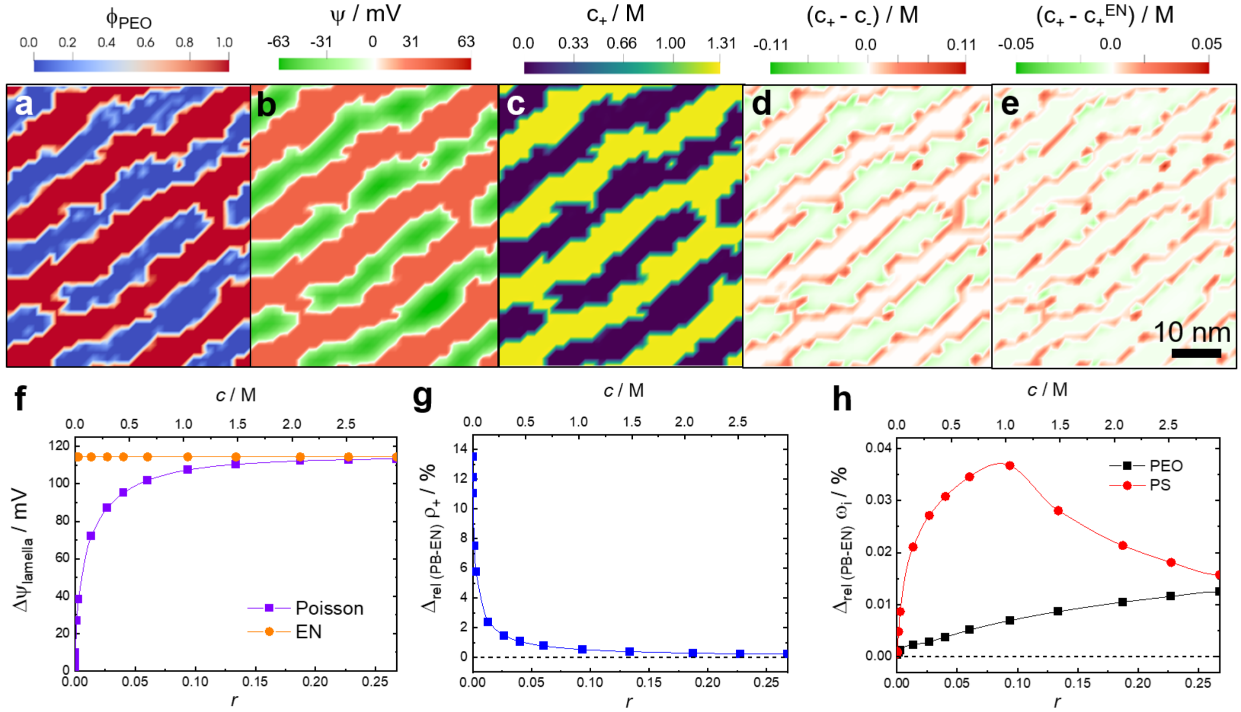

Figure S1: a-d. Color maps showing the local fraction of PEO segments (a), the electrostatic potential (in mV, 1 mV = 0.032 $k_\mathrm{B}T/e$ at T = 90ºC) (b), the molar concentration of cations (c) and difference between the molar concentration of cations and anions (d), predicted by the SCMF simulation without the EN approximation. e. Color map of the difference of the molar concentration of cations predicted without (c_+) and with (c_+^{EN}) the EN approximation. f-h) Average relative error of using the EN approximation on the molar concentration of cations (f), the electrostatic potential (g) and the field ω_α for a segment of type α (h).

between the domains. The EN approximation underestimates the Li^+ concentration on the PEO side of the interface and overestimates it at the PS side, as expected from the fact that the nonapproximate solution predicts that the Li^+ concentration at the PEO side is higher than that of $TFSI^-$, see Figure S1c.

Figure S1f-h quantify the average error produced by the EN approximation for different values of the Li^+:EO ratio, r. In order to isolate the effect of the calculation of the electrostatic potential, the results in these panels were calculated by fixing the distribution of the polymer segments to that shown in panels a-e. Then, the electrostatic potential was obtained either by solving the Poisson Equation (Equation 12) or by using the EN approximation, Equation 22. Figure S1f shows the electrostatic potential difference, $\Delta\psi_{\text{PEO}-\text{PS}}$, between the PEO and PS domains as a function of r. In the EN approximation, this potential difference only depends on the dielectric constants of both blocks and the ionic radii, Equation 22, and, therefore, it is independent of r. The nonapproximate solution converges to the EN prediction for large r. As r decreases, the electrostatic potential in the system should converge to that of the salt-free material, where there is no difference in the electrostatic potential between the two microphases. One can arrive at the same conclusion by recalling that the characteristic length scale of the double layer, the Debye length, increases with the decreasing salt concentration. For decreasing r, the double layer becomes commensurable with the thickness of a domain and, therefore, the electrostatic potential no longer reaches the bulk values predicted by Equation 22. In summary, the EN approximation fails to properly predict $\Delta\psi_{\text{PEO}-\text{PS}}$ for $r < 0.08 - 0.1$.

Panels g and h of Figure S1 plot the average error introduced by the EN approximation in the concentration profile of Li^+ ions and in the ω_α fields that determine the interaction of the polymer species with their environment, see Equation S1. In these plots, we defined the average error of using the EN approximation for an observable $\mathcal{O}$ as

$$\Delta_{\text{rel}}^{\text{EN}} f = \frac{\left\langle \left| \mathcal{O}\left(\mathbf{r}\right) - \mathcal{O}^{\text{EN}}\left(\mathbf{r}\right) \right| \right\rangle}{\max\left(\mathcal{O}\left(\mathbf{r}\right)\right) - \min\left(\mathcal{O}\left(\mathbf{r}\right)\right)} \tag{S9}$$

where $\mathcal{O}$ and $\mathcal{O}^{\text{EN}}$ are the observables predicted without and with the EN approximation, respectively, for fixed density profiles of the polymer species.

The relative error introduced by the EN approximation in the Li^+ molar concentration,c_+, see Figure S1g, sharply increases below $r < 0.05$. It is important to note that while we fixed here the morphology of the system to a lamellar structure, in reality the system will be disordered for small values of r (*e.g.*, for 90ºC, the ODT is predicted for $r \sim 0.08$, see Figure 1 in the main text). In other words, under the conditions where the EN approximation starts to fail for the lamella phase, ψ and c_+ will become homogeneous.

We finally address the effect of the EN approximation on the ω_α fields that control the interaction of the polymer species with their environment, Figure S1h. The first important observation is that the mean error is very small, < 0.04 %, for the whole range of r, which indicates that although the EN fails to properly capture the electrostatic potential at low r, this failure does not affect the polymer dynamics. The relative error in ω_α vanishes at $r \to 0$. This is expected because under those conditions the material behaves as the salt-free block copolymer, which is completely neutral. Increasing r leads to an increase in the relative error. The relative error peaks around $r = 0.1$ for ω_{PEO} because the EN becomes a good approximation for large r.

In summary, the EN approximation properly describes the inhomogeneities of electrostatic potential and ion concentration in self-assembled microphase-separated domains for large r. In principle, it is a bad approximation close to and below the ODT, however under those conditions the heterogeneities of electrostatic potential and ion concentration are small and, therefore, the errors introduced by using the EN are inconsequential. Even more important, the EN approximation properly predicts the ω_α fields under all conditions and, therefore, produces the same polymer dynamics and self-assembly behavior as the nonapproximate solution of the Poisson equation.

Boundary conditions for the generalized diffusion equations

Equation 18 does not depend explicitly on ψ. Since applying a constant cell potential fixes $\Delta V = \psi\left(z = L\right) - \psi\left(z = 0\right)$, it is necessary to relate this boundary condition to one expressed in terms of ion concentrations, as it is required by Equation 18. Let us consider a simplified 1D description of the problem, where all functions depend only on z, the axis perpendicular to the electrodes. In Li batteries, the anode and cathode are blocking electrodes for the TFSI^- anions, thus in the steady-state (*i.e.*, a fully polarized cell), the anion current vanishes at all z positions,

$$J_-(z) = -\frac{D_-\rho_-(z)}{k_\mathrm{B}T}\left[k_\mathrm{B}T\frac{\mathrm{d}}{\mathrm{d}z}\ln\rho_-(z)\lambda_\mathrm{T}^3 + \frac{\mathrm{d}}{\mathrm{d}z}z_-e\psi\left(z\right) + \frac{\mathrm{d}}{\mathrm{d}z}u_{\mathrm{Born},-}(z)\right] = 0 \tag{S10}$$

Therefore,

$$-z_-\frac{\mathrm{d}}{\mathrm{d}z}e\psi(z) = \frac{\mathrm{d}}{\mathrm{d}z}k_\mathrm{B}T\ln\rho_-(z) + \frac{\mathrm{d}}{\mathrm{d}z}u_{\mathrm{Born},-}(z) \tag{S11}$$

Integrating between the surfaces of both electrodes, assuming that the dielectric constants on those surfaces (and thus $\nabla u_{\mathrm{Born},-}$) are equal and rearranging, results in:

$$\frac{\rho_-(L)}{\rho_-(0)} = \exp\left(-\frac{z_-e[\psi(L)-\psi(z)]}{k_\mathrm{B}T}\right) = \exp\left(-\frac{z_-e\Delta V}{k_\mathrm{B}T}\right) \tag{S12}$$

Therefore, fixing ΔV results in a fixed ratio of salt concentration on the surfaces of the electrodes, which set up the boundary condition for Equation 18.

Calculation of the current density

The total current density circulating through the cell, j ,can be obtained by the integral of the Li^+ flux:

$$j = e\int \mathbf{J}_+(x,y,z)\hat{\mathbf{n}}_z dxdy \tag{S13}$$

This calculation can be performed at any value of z because in steady-state conditions j is independent of z (in the practice, we calculate j at both electrodes and check that the resulting values are the same).

Morphology of PEO-PS + TFSI in the absence of dielectrophoretic forces

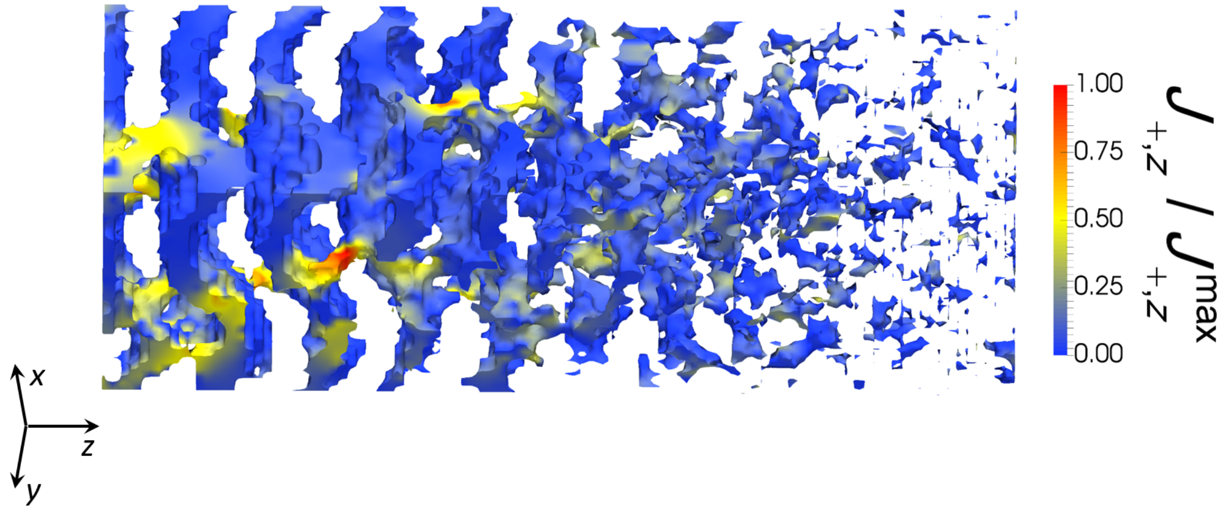

Figure S2: Same as Figure 6 in the main text, but setting the dielectrophoretic contribution to ω_α ($\omega_{\alpha,\mathrm{el}}$, Equation S6) to zero. The simulation time was 59.3 τ_R.

Current transient for the formation of the bicontinous morphology

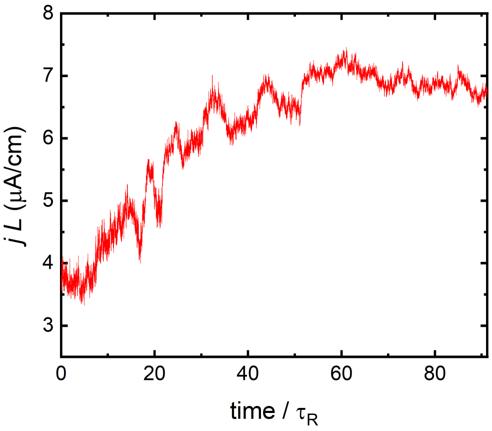

Figure S3: Current-length product, jL, *vs* simulation time for the results in Figure 6 in the main text. A potential jump from $\Delta V = 0$ to $\Delta V = 42.3$ mV was applied at $t = 0$.

References

(1) Welling, U.; Müller, M.; Shalev, H.; Tsori, Y. Block Copolymer Ordering in Cylindrical Capacitors. *Macromolecules* **2014**, *47*, 1850–1864.

(2) Dreyer, O.; Schneider, L.; Radjabian, M.; Abetz, V.; Müller, M. Evaporation-Induced Self-Assembly of Diblock Copolymer Films in an Electric Field: A Simulation Study. *Macromolecules* **2023**, *56*, 6880–6890.

(3) Nakamura, I.; Balsara, N. P.; Wang, Z.-G. Thermodynamics of Ion-Containing Polymer Blends and Block Copolymers. *Physical Review Letters* **2011**, *107*, 198301.

(4) Nakamura, I.; Wang, Z.-G. Salt-Doped Block Copolymers: Ion Distribution, Domain Spacing and Effective χ Parameter. *Soft Matter* **2012**, *8*, 9356–9367.

(5) Ohshima, H.; Ohki, S. Donnan Potential and Surface Potential of a Charged Membrane. *Biophysical Journal* **1985**, *47*, 673–678.
